# Association of Functional Gene Variants in DYSF–ZNF638, MTSS1 and Ferroptosis-Related Genes with Multiple Sclerosis Severity and Target Gene Expression

**DOI:** 10.3390/ijms26114986

**Published:** 2025-05-22

**Authors:** Tamara Djuric, Ana Djordjevic, Jovana Kuveljic, Milan Stefanovic, Evica Dincic, Ana Kolakovic, Maja Zivkovic

**Affiliations:** 1 Laboratory for Radiobiology and Molecular Genetics, VINČA Institute of Nuclear Sciences—National Institute of the Republic of Serbia, University of Belgrade, 11000 Belgrade, Serbia; ana.djordjevic@vin.bg.ac.rs (A.D.); jovana@vin.bg.ac.rs (J.K.); milanst@vin.bg.ac.rs (M.S.); anakolakovic@vin.bg.ac.rs (A.K.); majaz@vin.bg.ac.rs (M.Z.); 2 Clinic for Neurology, Military Medical Academy, 11000 Belgrade, Serbia; evica.vma@gmail.com; 3 Medical Faculty, Military Medical Academy, University of Defense, 11000 Belgrade, Serbia

**Keywords:** multiple sclerosis, MS severity, ferroptosis-related genes, gene variants, mRNA expression, lipid peroxidation and iron metabolism products

## Abstract

Multiple sclerosis (MS) is a chronic inflammatory, neurodegenerative disease with yet-unresolved mechanisms of progression. To address MS severity and neurological deficits, we analyzed seven potentially functional genetic variants and their haplotypes in 845 MS patients. Based on our previous results of targeted RNAseq on ferroptosis-related genes in distinctive MS phenotypes, we selected putative regulatory variants in the top three DEGs (*CDKN1A*, *MAP1B* and *EGLN2*) and investigated their association with gene expression, plasma/serum parameters and disease severity (EDSS, MSSS, gARMSS). The study included 604 patients with relapsing–remitting (RR) and 241 with progressive (P) MS. The variants *CDKN1A* rs3176326 and rs3176336, *EGLN2* rs111833532, *MAP1B* rs62363242 and rs1217817 with the previously reported *DYSF-ZNF638* locus rs10191329, and *MTSS1* rs9643199 were genotyped using TaqMan^®^, and the HLA-DRB1*15:01 status was also determined. Significant association of the rare *MAP1B* rs62363242 allele with PMS in females, independent of HLA-DRB1*1501, was found. The A allele-containing genotypes were associated with molecular components of iron metabolism. *CDKN1A* haplotypes were significantly associated with *CDKN1A* mRNA levels in RRMS and SPMS patients. *RAB4B-EGLN2* locus rs111833532 and *DYSF-ZNF638* locus rs10191329 showed significant associations with EDSS, MSSS and gARMSS. We detected haplotypes associated with the expression of *CDKN1A*, a part of the p53-p21 axis known to affect T cell activation/proliferation. *RAB4B-EGLN2,* an oxygen sensor and critical regulator of the response to hypoxia, variant rs111833532, along with DYSF-ZNF638 locus rs10191329, was associated with clinical severity. The indicated, novel, sex-specific association of *MAP1B* rs62363242 with the course of MS remains to be validated in larger studies.

## 1. Introduction

Multiple sclerosis (MS) is chronic inflammatory and demyelination-mediated neurodegenerative disease. It is a leading cause of neurological disability in adults that is not caused by trauma. Research on MS genetic risk factors was exponentially improved by a genome-wide association study (GWAS) that identified approximately 250 variants residing outside of the human leukocyte antigen (HLA) loci, and robustly confirmed the association of MS with HLA variants, among which HLA-DRB1501 allele A carries the highest genetic risk, with the strongest association [1]. In addition, the definition of biological pathways and processes that are relevant for MS, beyond those related to immune response [2], was and is among the primary research goals. MS is characterized by acute inflammation during disease relapses, chronic inflammation which overlaps with neurodegeneration, and often tissue- and cell-specific responses; thus, the complexity of its etiology requires further expansion of understanding of the roles and relations of many genes, processes and their associations with the clinical expression of the disease [3].

Still, the major challenge lies in resolving both genetic and environmental factors related to the disease’s severity and progression. All the results obtained from genetic association studies suggest that different biological processes and pathways contribute to MS onset and MS severity [4,5]. Two recent GWASs focused on defining the genetic background of MS severity [2,6] reported only a few variants that reached the GWA significance threshold. Some of them failed to be validated in studies that followed [7,8]. One of the biological pathways that is suggested to have a role in MS severity is ferroptosis. This iron-dependent [9,10], regulated cell death is proposed to affect both inflammation and neurodegeneration in the CNS [11,12].

We recently conducted research on ferroptosis-related genes by targeted RNAseq in PBMCs from highly distinctive MS phenotype groups with regard to disease severity [13]. Our work demonstrated 26 differentially expressed genes (DEGs), out of 138 genes studied, between mild relapsing–remitting (RR) and severe secondary progressive (SP) MS patients. The top three DEGs were cyclin dependent kinase inhibitor 1A (*CDKN1A*), egl-9 family hypoxia inducible factor 2 (*EGLN2*) and microtubule associated protein 1B (*MAP1B*) [13]. We aimed to search for and select common gene variants that are potential transcriptional regulators of these genes. The majority of such common variants do not affect protein sequences, and are located in non-coding introns or gene-flanking regions, including promoters/enhancers, which represent about 80% of the human genome [14]. Through an integrative approach involving extensive datamining of multiple public databases (FIVEx, GTEx, RegulomeDB, ENSEMBLE, HaploReg, GWAS Catalogue, GnomAD) and a literature search, we selected five gene variants proposed to be expression quantitative trait loci (eQTLs) for the top three DEGs: for *CDKN1A* rs3176326 and rs3176336, for *EGLN2* rs111833532 from the *RAB4B-EGLN2* locus and for *MAP1B* rs62363242 and rs1217817. In addition, two more gene variants from the above-mentioned GWASs that have been associated with MS severity [2,6] were chosen and included in the study: rs10191329 from the *DYSF–ZNF638* locus [6] and rs9643199 from the *MTSS1* gene [2]. Of note is that all of the patients included in the study were genotyped for HLA-DRB1*15:01.

The aims were to analyze whether the selected seven variants are associated with MS severity, whether they affect the mRNA expression levels of the top three ferroptosis-related DEGs (*CDKN1A*, *MAP1B* and *EGLN2*) in RRMS and SPMS patients, and whether they are associated with MS neurological deficit and severity parameters (EDSS, MSSS, gARMSS), taking into account the existing haplotypes. In addition, we analyzed their possible association with measured molecular components of ferroptosis-related processes, such as lipid peroxidation and iron metabolism products, in the plasma/serum of MS patients.

## 2. Results

### 2.1. Genetic Association Analysis of Selected Variants with MS Disease Severity

The main characteristics of the study groups involved in the genetic association analysis are presented in Table 1. Age, disease duration, EDSS, MSSS and gARMSS were significantly different between the distinct MS phenotypes, RRMS and PMS, which was expected. The percentage of males and females between groups was not significantly different.

The gene variant *MAP1B* rs62363242 showed significantly different genotype frequency distribution between RRMS and PMS patients in females only. The *MAP1B* rs62363242 rare A allele containing genotypes (*p* = 0.03), as well as the A allele itself (*p* = 0.03), were significantly more frequent in PMS female patients than in RRMS females (Table 2), showing the significant genotype x sex effect on disease course (*p* = 0.026). The power of the study for this association was 62%. The rare A allele-containing genotypes were associated with PMS course in females with an adjusted OR = 1.56, 95%CI (1.06–2.29) and *p* = 0.02, independent of the presence of the HLA-DRB1*15:01 rs3135388 A allele. The *MAP1B* rs62363242 A allele showed a significant association with PMS, with an OR = 1.35, 95CI% (1.02–1.79) and *p* = 0.03. Another six investigated gene variants—*RAB4B-EGLN2* rs111833532, *CDKN1A* rs3176326 and rs3176336, *MAP1B* rs1217817, *DYSF–ZNF638* locus rs10191329 and *MTSS1* rs9643199—did not show significant associations with the progressive MS phenotype (Table 2).

### 2.2. Effects of CDKN1A rs3176326 and rs3176336, RAB4B-EGLN2 rs111833532, and MAP1B rs62363242 and rs1217817 on Their mRNA Relative Expression Levels in PBMCs of RRMS and SPMS Patients

**Figure 1:**
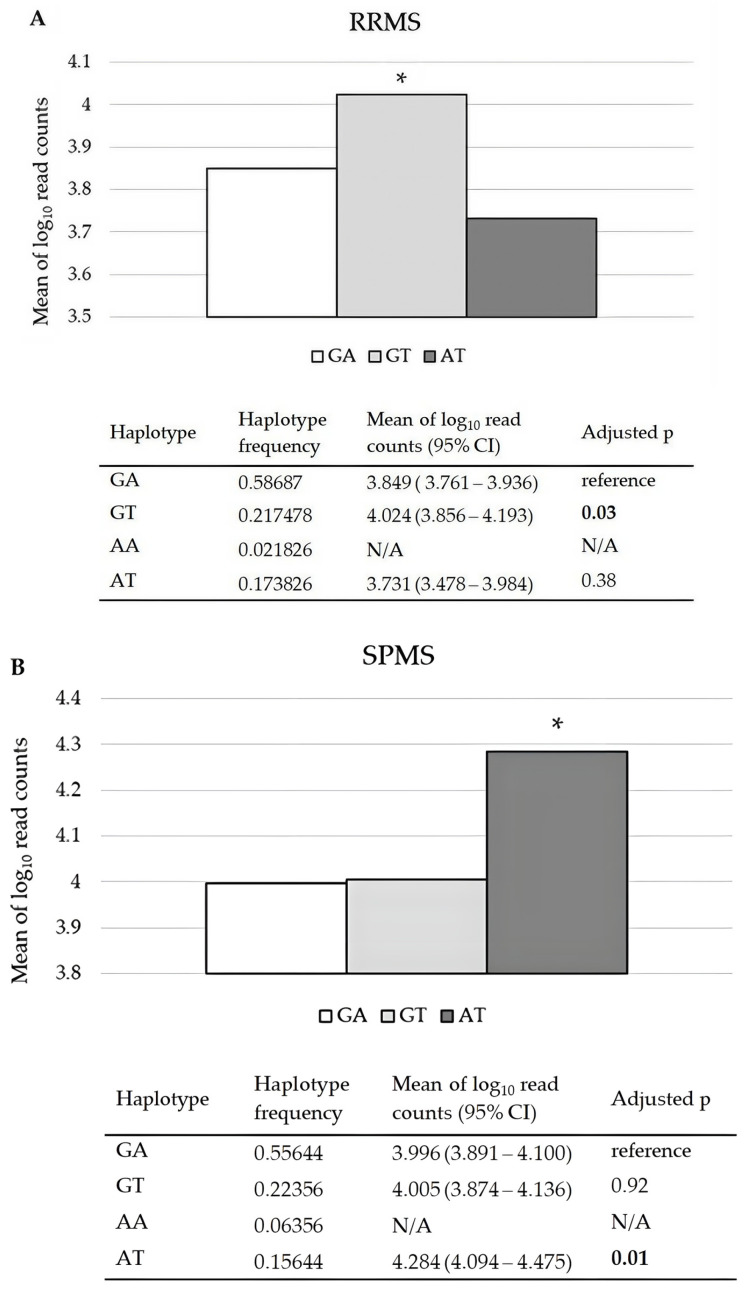
Effects of CDKN1A haplotypes inferred from rs3176326 G/A and rs3176336 A/T on relative CDKN1A mRNA levels in PBMCs of (**A**) RRMS and (**B**) SPMS patients. The order of alleles in haplotypes represented in the figure legends is as follows: in the first line is the rs3176326 G or A allele, and in the second line is the rs3176336 A or T allele. The haplotype GA was set as a reference haplotype by Thesias software. *—adjusted *p*, the association was adjusted based on sex; CI—confidence interval; N/A—not applicable, the results regarding the haplotype AA were not presented due to low frequency in the studied groups.

*CDKN1A* haplotypes inferred from rs3176326 G/A and rs3176336 A/T were significantly associated with relative *CDKN1A* mRNA levels in RRMS and SPMS patients, independently of sex (Figure 1A,B). The haplotype GT was associated with significantly higher *CDKN1A* mRNA levels compared to the reference haplotype GA (set by the Thesias 3.1 software) in PBMCs of RRMS patients (*p* = 0.03). In SPMS patients, the haplotype AT (both rare alleles) was associated with higher *CDKN1A* mRNA levels in PBMCs compared to the reference GA (*p* = 0.01), also independently of sex. The results regarding haplotype AA were not presented due to low frequency in the studied groups (the study groups included 24 RRMS and 24 SPMS). *RAB4B-EGLN2* rs111833532 and *MAP1B* rs62363242 and rs1217817 (either solely or in haplotype) were not associated with the *EGLN2* and *MAP1B* relative mRNA levels in PBMCs of RRMS and PMS patients.

### 2.3. Associations of Investigated Gene Variants with MS Neurological Deficit and Severity Parameters (EDSS, MSSS, gARMSS)

Since *RAB4B-EGLN2* locus rs111833532 I/D, according to the allele D dominant model, showed univariate association with all three neurological deficit and severity parameters, EDSS, MSSS and gARMSS, in RRMS patients, we performed multiple linear regression analysis for EDSS, MSSS and gARMSS in RRMS patients with a model that included the following: sex, disease duration, age at MS onset, rs111833532 I/D according to allele D dominant model, and presence of the HLA-DRB1*15:01 rs3135388 risk A allele. Disease duration, age at onset and the rs111833532 D allele, according to the dominant model, were significantly associated with all three investigated parameters (Table 3).

*DYSF-ZNF638* locus rs10191329 C/A, according to the A allele dominant model, showed significant association with higher EDSS, MSSS and gARMSS in male PMS patients. So, we performed the multiple linear regression analysis for EDSS, MSSS and gARMSS in male PMS patients with model that includes: disease duration, age at MS onset, *DYSF-ZNF638* locus rs10191329 C/A according to A allele dominant model and presence of HLA-DRB1*15:01 rs3135388 risk A allele (Table 4). The only significant predictor for EDSS in the model was *DYSF-ZNF638* rs10191329 allele A containing genotypes (*p* = 0.03), the significant predictor for MSSS was only disease duration (<0.001), while for gARMSS significant predictors were disease duration, the age of onset and *DYSF-ZNF638* rs10191329 allele A containing genotypes (0.01, <0.001 and 0.04, respectively) (Table 4).

One more association was found within the PMS group, but in females only. *MTSS1* rs9643199 wild-type GG homozygotes had a significantly higher MSSS compared to rare allele A-containing genotypes (7.00 ± 1.83 vs. 6.14 ± 1.98, respectively, *p* = 0.01).

The *CDKN1A* GT haplotype, inferred from gene variants rs3176326 G/A and rs3176336 A/T, compared to the reference haplotype GA, was associated with a higher EDSS score in RRMS patients (2.78 (2.49–3.07) vs. 2.35 (2.17–2.52), respectively, *p* = 0.02).

### 2.4. Association of Investigated Gene Variants with Circulatory Molecular Indicators of Processes Associated with Ferroptosis: Lipid Peroxidation (MDA, 4-HNE and HEL), GSH-Related Antioxidant Defense (GSH, GSSG and GPX4) and Iron Metabolism (Free Iron, Transferrin and Ferritin), in MS Patients

From the investigated gene variants, *RAB4B-EGLN2* rs111833532 rare allele D-containing genotypes (II vs. ID + DD) showed a trend toward association with higher plasma 4-HNE in MS patients overall (0.06), but statistical significance was achieved in the plasma of PMS patients (*p* = 0.04) (Table 5). *MAP1B* rs62363242 rare allele A-containing genotypes (GG vs. GA + AA) were significantly associated with iron metabolism parameters—lower free iron (*p* = 0.03) and higher transferrin (*p* = 0.03)—in the serum of PMS patients. There was also a trend toward a lower level of the third measured parameter, ferritin (*p* = 0.08), within the same group of patients (Table 5).

Since we genotyped the whole study group for HLA-DRB1*15:01 rs3135388 G/A to adjust for the significant association of investigated variants with progressive MS disease course, we analyzed whether this variant could have effects on circulatory molecular indicators of processes associated with ferroptosis. HLA-DRB1*15:01 rs3135388 risk A allele-containing genotypes were significantly associated with higher levels of HEL (hexanoyl-lys adduct) in the serum of MS patients overall (*p* = 0.02) (Table 5).

## 3. Discussion

In this study, we investigated gene variants proposed to be regulatory for *CDKN1A*, *EGLN2* and *MAP1B*, the top three DEGs derived from our previous research of 138 ferroptosis-related genes between RRMS and SPMS, and which have been found to be associated with MS severity in recent GWASs [2,6]. We have shown that the *MAP1B* variant rs62363242 is associated with progressive MS disease course in females, and this association is independent of HLA-DRB1*1501 genotypes. There was a significantly higher frequency of *MAP1B* rs62363242 rare allele A- and A allele-containing genotypes in PMS females compared to RRMS females. In our previous work, *MAP1B* mRNA was significantly more highly expressed in PBMCs of SPMS compared to RRMS patients [13]. *MAP1B* rs62363242 is proposed to be an eQTL for this gene in different parts of the brain [15]. According to the databases, the rs62363242 rare allele A is related to higher expression of *MAP1B* mRNA. The *MAP1B* gene encodes a protein that belongs to the microtubule-associated protein family that is important for axonal growth and synapse maturation during brain development [16,17]. Nevertheless, evidence from animals shows that it is also highly expressed in the adult brain, having a role in axonal plasticity and regeneration [18,19]. In *MAP1B*-deficient mice, the myelin sheets over the axons had reduced thickness and, in general, there were a reduced number of large myelinated axons [20]. Another study showed that MAP1B accumulation led to progressive cell death [21], and that the full-length *MAP1B* transcript resulted in the acceleration of neuronal death in rats [22]. Still, there is no straightforward conclusion about the possible protective or detrimental role of *MAP1B* in the adult brain. In this study, we found an association of *MAP1B* rs62363242 A allele-containing genotypes with lower serum iron and higher transferrin levels, while there was a trend towards an association with ferritin, in PMS patients. In a recent study that investigated the effect of iron deficiency on embryo development in iron-deficient animals, differential expression of genes that are involved in regulation of the cell cycle, cell growth and proliferation was observed. The researchers found a marked increase in the expression of *MAP1B*, and suggested that this up-regulation may result in increased apoptosis in iron deficient embryos [23]. Multiple studies have indicated that the serum iron levels of MS patients were lower than or equal to those of controls, whereas transferrin levels were increased compared to healthy controls [24]. This ratio is similar to our results, but in PMS patients only, and according to different *MAP1B* genotypes. Altogether, these results suggest that background mechanisms connecting the ferroptosis-related gene *MAP1B* and molecular components involved in iron metabolism need further investigation.

The *CDKN1A* gene variants rs3176326 and rs3176336 are proposed to be functional according to Regulome DB [25]. CDKN1A, a part of the p53-p21 axis known to affect the activation/proliferation of T cells, has multiple roles in physiological and pathological states. Besides its role in cell cycle regulation, it has prominent roles in apoptosis, transcription and differentiation [26]. Recent findings relate CDKN1A to apoptosis and remyelination in different brain cells. High expression of the *CDKN1A* gene was found in microglia nodules compared to stroke nodules in donors’ brain tissue post mortem [27]. It was shown that chronic microglial inflammatory activity in MS damages myelin and disrupts axonal and synaptic activity [28]. CDKN1 is a molecule with a recently underlined multifaceted role in both DNA damage repair and senescence, roles that go beyond its primary actions in the regulation of the cell cycle [29]. It is required for the regulation of response to DNA damage, as well for establishing oligodendrocyte differentiation, which is relevant for demyelination and neurodegeneration in MS. On the other hand, it has been noted that deletion of the p21 pathway ameliorates microglial inflammation and neurodegeneration [30]. Data on MS patients are still scarce, and our previous work showed higher expression of *CDKN1A* mRNA in SPMS compared to RRMS patients [13]. In the current study, we found higher expression of *CDKN1A* mRNA in both MS groups, according to haplotypes inferred from the gene variants rs3176326 G/A and rs3176336 A/T. The haplotype bearing the two rare alleles AT in SPMS patients, and the haplotype GT in RRMS patients, were associated with higher *CDKN1A* mRNA in PBMCs compared to the reference GA haplotype, independently of sex. Moreover, the GT haplotype was associated with a higher EDSS score in RRMS patients. In both groups of patients, the rs3176336 T allele was in a haplotype associated with higher *CDKN1A* mRNA expression. This variant has been proposed to be a splicing quantitative trait locus (sQTL) for CDKN1A in EBV-transformed lymphocytes and whole blood, with the rs3176336 T allele having the highest intron excision ratio [15]. It is known that genomic loci which are defined as eQTLs may share sQTL signals, since alternative splicing affects gene expression levels [31]. It seems that role of CDKN1A in MS is significant, but the mechanisms are still not well studied.

The *RAB4B-EGLN2* rs111833532 I/D variant showed significant association with all three parameters of neurological deficit and disability—EDSS, MSSS and gARMSS—in RRMS patients in this study. This variant was proposed to be an eQTL for *EGLN2* in blood, T cells and B cells, but we did not find it to have a significant effect on the *EGLN2* mRNA level in PBMCs of RRMS or SPMS patients. Moreover, this variant has been proposed as an mQTL independent of the effect of other low-frequency or rare variants [32], and according to the HaploReg database, there is no gene variant that is in LD r^2^ > 0.8 with it. Indeed, this variant affects the DNA methylation patterns of CpG sites in the region at different life stages in humans [33]. Since MS is a disease that combines the effects of both genetic and environmental risk factors, epigenetic mechanisms are likely to be involved in its onset and progression. A recent study validated earlier findings that methylation was correlated with the expression of the HLA-DRB1*1501 allele [34], and highlighted that methylation alone, apart from the MHC II region, was a stronger discriminator of MS than previously described known genetic risk factors [35]. The authors also found that the most prominent methylation differences in MS occur predominantly in B cells and monocytes. There are no data on the cell-specific mQTL potential of rs111833532, but it acts like an eQTL for EGLN2 in B cells, whereas the deletion allele has higher expression [36]. We can speculate that if methylation is one of the disease onset mechanisms, it could also be the mechanism or part of the mechanism of disease progression, meaning that variants proposed as mQTLs could affect the progression and severity of MS. A novel parameter that improves the assessment of disease progression and severity, gARMSS, takes into account the patient’s age at the time of assessment of EDSS [37]. The association of *RAB4B-EGLN2* rs111833532 D allele-containing genotypes goes in the same direction for all three parameters of disease severity, i.e., they are associated with higher EDSS, MSSS and gARMSS scores in RRMS patients. Further, the same genotypes have been associated with higher levels of 4-HNE in the plasma of PMS patients. The aldehyde 4-HNE is a product of lipid peroxidation, which has been shown to increase during ferroptosis [38]. It has been investigated in neurodegeneration, including in MS [39,40]. Formation of 4-HNE-protein adducts contributes to neurodegeneration [41], which is a key feature of PMS. We propose that this gene variant should be further validated and replicated in larger sample groups, and most of all, we propose a deeper investigation of the mechanisms by which EGLN2, an oxygen sensor and critical regulator of the response to hypoxia, could affect neurological deficit and disability in MS.

One of the two recent GWASs that investigated the genetics of MS progression pointed out that *DYSF-ZNF638* rs10191329 A allele carriers had faster disability progression [6]. In this study, we found a significant association of the same genotypes (A allele carriers) with higher EDSS and gARMSS, but only in male patients with PMS. It was shown that the risk allele A has a dosage effect on worsening of the injury at key brain locations, e.g., a higher number of cortical lesions and lesions in the brainstem, both of which are determinants of progression [42,43]. A recent study associated this allele with a higher percentage of brain atrophy in RRMS patients, but not with EDSS [44]. Other recently published studies failed to replicate the association of *DYSF-ZNF638* rs10191329 with longitudinal ARMSS or MSSS [7]. The debate on the mechanistic background of this variant and which target gene it regulates is still going on. According to databases, rs10191329 is an eQTL for ZNF638 in blood, T cells and B cells [35], an eQTL for lncRNA DYSF-2 in T cells, Th17 memory and naive B cells [45]. One of its related pathways is nervous system development, and gene ontology annotations related to this gene include nucleic acid binding and RNA binding [46]. It is also an sQTL for ZNF638, but not in tissues that are involved in MS development or progression [15]. There is not enough experimentally evidenced data from scientific research to clarify whether this association is the cause or the consequence of the PMS phenotype. Further functional studies are needed to reveal its plausible mechanistic background for MS severity.

The second GWAS that analyzed the genetics of MS severity found a sex-specific association of the *MTSS1* rs9643199 A allele with longitudinal MSSS in females [2]. In our study, *MTSS1* rs9643199 wild-type GG homozygotes had significantly higher MSSS compared to rare allele A-containing genotypes in females within the PMS group. The discrepancy in the results reflects different study designs. We analyzed the association with cross-sectional MSSS values, whereas in the study by Jokubaitis et al. [2], the phenotype of interest was longitudinal MSSS. Nevertheless, further validation in a larger group is inevitable before any conclusion can be drawn.

We also genotyped our study population for rs3135388, a tag SNP for the HLA DRB1*1501, a well-known susceptibility risk factor for MS with the greatest predictive power of weighted genetic risk scores for MS [1]. We stratified our patients by the existence of the rs3135388 risk A allele, and adjusted most of the associations for its presence. We found that this susceptibility marker was significantly associated with higher levels of HEL in the serum of MS patients overall. HEL is a novel early-stage lipid peroxidation indicator, and as such, a potential biomarker for ferroptosis-related processes. Lipid peroxidation is involved in the development of demyelination and neurodegeneration, characteristics of MS [47]. Therefore, the susceptibility marker for MS is also related to higher lipid peroxidation markers in the circulation of MS patients. To our knowledge, this is the first association of the HLA DRB1*1501 A allele with serum HEL levels, and as such, this needs validation and further investigation.

Although a considerable number of patients were included in the study, a constant limitation in such studies is the inclusion of an equal number of patients with relapsing–remitting and progressive disease. Moreover, the subgroup of patients in whom mRNA levels were measured by targeted RNAseq consisted of only 48 individuals, although the groups were homogeneous, which may be the reason why we did not find associations with variants besides *CDKN1A* haplotypes. Biological relevance for some of the variants still needs to be explored through additional experimental studies or studies on animal models.

In this study, we focused on datamining, selecting and analyzing gene variants with a strongly proposed functional capacity to regulate the expression of ferroptosis-related genes relevant to MS severity, and added those that have been associated with MS severity in recent GWASs. The results suggest the sex-specific association of MAP1B rs62363242 with progressive MS course. Furthermore, the significant effect of CDKN1A haplotypes on its’ gene expression in both RRMS and SPMS suggests that certain molecular processes may be relevant in both phases of MS, consistent with the recently adopted concept that further research needs to perceive this complex disease as a continuum, beyond common stratification by disease course [3]. The results, especially those of the previously unstudied associations, need to be validated and replicated in larger study groups and in different populations.

## 4. Materials and Methods

### 4.1. Subjects

The study group for genetic association analysis involved 845 unrelated patients from Serbia, 604 with RRMS and 241 with PMS. The PMS patients included 209 patients with secondary progressive (SP) MS and 32 with primary progressive (PP) MS. The subgroup of patients in whom possible associations of targeted gene variants with circulatory molecular indicators of ferroptosis were analyzed consisted of 222 patients overall, comprising 153 with RRMS, 54 with SPMS and 15 with PPMS. The subgroup of patients for whom the mRNA levels were measured by targeted RNAseq comprised 48 (24 RRMS and 24 SP) MS patients [13]. A total of 623 patients were recruited from 2015 to 2018 during their visits to the Clinic for Neurology of Military Medical Academy, Belgrade; an additional 222 patients for whom circulatory molecular indicators of ferroptosis were measured were recruited from the Clinic for Neurology of Military Medical Academy, Belgrade, and the Neurology Clinic of University Clinical Center Nis, Nis, Serbia, from 2022 to 2023. The diagnosis of clinically definite MS was made according to revised McDonald criteria [48,49,50], and the course of disease (RRMS, SPMS and PPMS) was defined according to a clinical method [51,52]. None of the patients had relapse during the period of at least 30 days prior to the study enrollment. The questionnaire was fulfilled for each patient to provide accurate data on clinical and anthropometric parameters, which were determined at the time of peripheral blood sample collection. For the patients, overall anthropometric parameters included age and sex. Clinical parameters encompassed disease onset age, disease duration, expanded disability status scale (EDSS), multiple sclerosis severity score (MSSS) and age-related global multiple sclerosis severity (gARMSS) score. The clinical severity of the disease was assessed by using the EDSS score as a measure of neurological disability [53]. The progression of disability was determined by the MSSS, which corrects the EDSS for disease duration [54]. For every participant, we calculated the age-related global multiple sclerosis severity (gARMSS) score [37] based on the EDSS score and age at the time of assessment. gARMSS offers a more versatile tool for comparing EDSS-based MS severity between groups. gARMSS presents the ARMSS scores derived from the EDSS in patients aged 18 to 75 years [37]. The ARMSS score was calculated by an online tool (available at https://aliman.shinyapps.io/ARMSS/, accessed on 15 January 2024; [55]).

A detailed description of the subgroup with circulatory biochemical analysis was given in our previous paper [39], as well as of the patients in whom expression of target genes was investigated [13].

The MMA Ethics Committee approved the study (Decision from 25 February 2010, and Decision No 6/2020, date of approval: 4 August 2020), and each participant gave their written informed consent for participation in the study.

### 4.2. Selection of Genes and Gene Variants

According to our previously published results [13], considering a targeted mRNA sequencing panel made of 138 ferroptosis-related genes, we selected five variants from the top three differentially expressed genes (DEGs) between RRMS and SPMS: *CDKN1A, EGLN2* and *MAP1B*; and, according to the literature search including the two most recent GWASs of multiple sclerosis severity, we selected a variant in the *DYSF-ZNF638* locus [6] and a variant in the *MTSS1* gene associated with MS severity in women [2]. The selected variants were chosen with the regard to their potential functional effect on the expression of the target gene, and/or high-quality published results showing their association with MS disease severity.

Gene variant selection was performed by integration of multiple criteria: (1) a minor allele frequency (MAF) > 0.1 in the European non-Finnish population (available at https://gnomad.broadinstitute.org/, assessed on 9 January 2024; [56]); (2) categorization as an eQTL for the gene of interest in the organ/tissue of interest (blood, PBMC, lymphocytes, brain) in the public databases FIVEx: eQTL browser (available at https://fivex.sph.umich.edu/, assessed on 9 January 2024; [57]) and/or GTEx (available at https://gtexportal.org, assessed on 9 January 2024; [58]); (3) variants were prioritized that had a high probability of being functionally important in RegulomeDB (likely to affect binding and linked to expression of a gene target) (available at https://regulome.stanford.edu/regulome-search, assessed on 9 January 2024; [59]), so all the selected variants, except those taken from the GWASs, had a RegulomeDB score of no lower than 1f; (4) GWAS gene variant–trait associations for multiple sclerosis and MS severity using the GWAS Catalogue (available at https://www.ebi.ac.uk/gwas, assessed on 9 January 2024; [60]); (5) information on LD with variants in the region, positioning within enhancers and the effects they have on regulatory motifs, obtained using HaploReg version 4.2 (available at https://pubs.broadinstitute.org/, assessed on 9 January 2024; [61]).

Finally, the selected variants were *CDKN1A* rs3176326 and rs3176336, *EGLN2* rs111833532, *MAP1B* rs62363242 and rs1217817, *DYSF-ZNF638* locus rs10191329 and *MTSS1* rs9643199 (Table 6).

### 4.3. Genetic Analysis

Genomic DNA was extracted from whole peripheral blood samples by the phenol/chloroform extraction method. The gene variants rs3176326, rs3176336, rs111833532, rs62363242, rs1217817, rs10191329 and rs9643199 were detected by means of 7500 real-time PCR (Applied Biosystems, Waltham, MA, USA) using TaqMan^®^ technology for allele discrimination and the following assays: C__27846175_10, C___2969509_20, C_153275122_10, C__88636515_10, C___3076215_20, C__29954731_10 and C___2055045_10, respectively, purchased from and tested by Applied Biosystems, Waltham, MA, USA. Approximately 10% of the samples were randomly selected and genotyped a second time by a different investigator. The results of the repeated genotyping were 100% consistent with the results of the original genotyping. Each PCR reaction contained 120 ng of DNA. The HLA-DRB1*15:01 status was determined for all participants in the study (TaqMan^®^ assay C__27464665_30).

### 4.4. Isolation of PBMCs, Extraction of the Total RNA, Targeted RNASeq Library Synthesis and Targeted RNA Sequencing

A lymphocyte separation medium (PAA, GE Healthcare, Chicago, IL, USA) was used for the isolation of PBMCs from the peripheral blood samples. Total RNA was extracted from PBMCs using TRI Reagent (Ambion, Life Technologies, Austin, TX, USA). RNA samples were dissolved in nuclease-free water and stored at −80 °C. RNA concentration and purity were determined using a NanoDrop ND-1000 spectrophotometer (Thermo Scientific, Waltham, MA, USA). Normalized read counts depicting the expression of target genes (*CDKN1*, *MAP1B* and *EGLN2*) performed on the 24 RRMS samples and 24 SPMS samples obtained in our previous study [13]. In brief, synthesis of sequencing libraries was performed according to the AmpliSeq^TM^ Library PLUS for Illumina^®^, using an in-house-designed custom RNA panel of ferroptosis-related genes from PBMC RNA samples [13]. Pooled libraries were sequenced on an iSeq^TM^ 100 System with a run configuration of 2 × 151 bp. The obtained raw read counts were normalized during the DeSeq2 workflow [13] and exported for further data transformation and association analysis in the current study.

### 4.5. Quantification of MDA, 4-HNE, GPX4 and Glutathione in Plasma

Peripheral blood samples with EDTA were centrifuged at 1000× *g* for 15 min at +4 °C, within 30 min of collection. The supernatant plasma was collected and stored at −20 °C prior to use.

Quantification of human MDA (Malondialdehyde), 4-HNE (4-Hydroxynonenal) and GPX4 (Phospholipid hydroperoxide glutathione peroxidase) in the plasma of 222 patients was performed by FineTest^®^ ELISA kits (Wuhan Fine Biotech Co., Ltd., Wuhan, China), as previously described [39]. Oxidized (GSSG), reduced (GSH) and total (GSH + GSSG) glutathione was quantified in the plasma samples of 100 patients, 63 with RR and 37 with progressive course of MS, using a quantification kit for oxidized and reduced glutathione sourced from Sigma-Aldrich^®^ (Sigma-Aldrich, Merck KGaA, Darmstadt, Germany). Detailed methodology is described elsewhere [39].

### 4.6. Quantification of Hexanoyl-Lys Adduct (HEL), Iron, Transferrin and Ferritin in Serum

After the withdrawal, peripheral blood samples were allowed to clot at room temperature for 2 h, and then centrifuged at 1000× *g* for 20 min at +4 °C. Supernatant serum from 222 samples was collected and stored at −20 °C prior to use. Serum hexanoyl-lys adduct (HEL) was quantified using a JaICA Hexanoyl-Lys adduct (HEL) ELISA kit (Japan Institute for the Control of Aging (JaICA), Nikken SEIL Co., Ltd., Haruoka, Fukuroi, Shizuoka, Japan). The detailed procedure, according to the manufacturer’s instructions, has been previously described [39].

In 222 patients’ serum samples, the iron concentration (µmol/L) was determined by spectrophotometry, and transferrin (g/L) by immunoturbidimetry, using the URIT-8210 Automatic clinical chemistry analyzer (URIT Medical Electronic Co., Ltd., Shenzhen, China), and the ferritin serum concentration (ng/mL) was determined by immunoturbidimetry, using the AutoLumo A1000 Chemiluminescence Immunoassay System (Autobio Diagnostics Co., Ltd., Zhengzhou, China).

### 4.7. Statistical Analysis

The difference between categorical variables, genotype and allele frequency distributions and deviation from the Hardy–Weinberg equilibrium were calculated by chi-square tests. The strength of association between genotypes and phenotypes of interest was calculated by logistic linear regression and presented as an odds ratio (OR) with its 95% confidence interval (CI). The power of the study for the obtained associations of gene variants with phenotypes was calculated by PS—Power and Sample Size calculator [62]. Student’s T test or the Mann–Whitney U test was used to compare continual variables between two groups depending on their distribution: normal or skewed (respectively). Data were presented as the mean ± standard deviation (SD) in order to make them more meaningful for the readership. Multiple linear regression analysis was performed to analyze multiple predictors, including gene variants, for EDSS, MSSS and gARMSS in MS patients. The statistical analysis was performed using Statistica 8.0 software (StatSoft, Inc., Tulsa, OK, USA). Haplotype frequencies and the effects of *CDKN1A* gene variants on the *CDKN1A* mRNA level were estimated by the Thesias software, which implements the stochastic-EM (Expectation–Maximization) algorithm for haplotype analysis [63]. *CDKN1A* mRNA expression levels were presented as Log_(10)_-transformed read counts. According to the Thesias software manual, the results of haplotypes’ effect on relative mRNA expression, as well as on any continuous variables, are presented as expected phenotypic means for one dose of each haplotype, with their corresponding 95% confidence interval compared to the reference haplotype. The reference haplotype was set by the Thesias software, and represents the most frequent haplotype in the studied groups. In all tests, *p* < 0.05 values were considered significant.

## Figures and Tables

**Table 1:** Basic characteristics of the MS patients, divided with regard to MS severity into relapsing–remitting MS (RRMS) and progressive MS (PMS) groups, involved in the analysis of gene variants.

	RRMS *n* = 604	PMS *n* = 241	*p*
Age, years	38.86 ± 10.41	47.27 ± 9.93	<0.01 ^a^
Sex, f/m, %	0.61/0.39	0.64/0.36	0.53 ^b^
Disease duration, years	7.73 ± 5.83	14.07 ± 9.44	<0.01 ^c^
EDSS	2.47 ± 1.32	5.59 ± 1.56	<0.01 ^c^
MSSS	3.91 ± 2.35	6.52 ± 2.03	<0.01 ^c^
gARMSS	4.58 ± 2.23	7.17 ± 2.01	<0.01 ^c^

Continual parameters are presented as mean ± standard deviation (SD); RRMS—relapsing–remitting multiple sclerosis; PMS—progressive multiple sclerosis (SPMS + PPMS); SPMS—secondary progressive multiple sclerosis; PPMS—primary progressive multiple sclerosis; Age—age at blood sampling; EDSS—Expanded Disability Status Scale; MSSS—Multiple Sclerosis Severity Score; gARMSS—Age-Related Global MS Severity Score; a—Student’s T test; b—Chi-square test; c—Mann–Whitney U test. *p* values < 0.05 are considered statistically significant.

**Table 2:** Genotype and allele frequency distribution of investigated gene variants in RRMS and PMS patients.

Gene/Locus	Gene Variant	RRMS, n (%) n = 604	PMS, n (%) n = 241	*p*
***CDKN1A***	**rs3176326**			
	GG	375 (0.62)	152 (0.63)	
	GA	193 (0.32)	77 (0.32)	0.82
	AA	36 (0.06)	12 (0.05)	
	allele G/A	0.78/0.22	0.79/0.21	0.65
***CDKN1A***	**rs3176336**			
	AA	187 (0.31)	70 (0.29)	
	AT	290 (0.48)	125 (0.52)	0.51
	TT	127 (0.21)	46 (0.19)	
	allele A/T	0.55/0.45	0.55/0.45	1
***RAB4B-EGLN2***	**rs111833532**			
	II	191 (0.32)	75 (0.31)	
	ID	296(0.49)	130 (0.54)	0.25
	DD	117 (0.19)	36 (0.15)	
	allele I/D	0.56/0.44	0.58/0.42	0.46
***DYSF–ZNF638***	rs10191329 *			
	CC	453 (0.75)	171 (0.71)	0.27
	CA +AA	151 (0.25)	70 (0.29)	
	allele C/A	0.86/0.14	0.86/0.14	1
***MTSS1***	**rs9643199**			
	GG	321 (0.53)	132 (0.55)	
	GA	225 (0.37)	91 (0.38)	0.62
	AA	58 (0.10)	18 (0.07)	
	allele G/A	0.72/0.28	0.74/0.26	0.43
***MAP1B***	**rs1217817**			
	GG	78 (0.13)	34 (0.14)	
	GA	242 (0.40)	101 (0.42)	0.66
	AA	284 (0.47)	106 (0.44)	
	allele G/A	0.33/0.67	0.35/0.65	0.41
***MAP1B***	**rs62363242**			
	GG	284 (0.47)	106 (0.44)	
	GA	254 (0.42)	111 (0.46)	0.50
	AA	66 (0.11)	24 (0.10)	
	allele G/A	0.68/0.32	0.67/0.33	0.68
	**Females**			
	**rs62363242**	RRMS, n (%) n = 367	PMS, n (%) n = 155	
	GG	187 (0.51)	64 (0.41)	**0.03**
	AG + AA	179 (0.49)	91 (0.59)	
	allele G/A	0.71/0.29	0.65/0.35	**0.03**

*p*—Chi-square test; *p* values < 0.05 are considered significant. * *DYSF–ZNF638* rs10191329 genotypes are presented according to dominant model, since in PMS group there was no rare homozygote AA.

**Table 3:** Multiple linear regression analysis with predictors of EDSS, MSSS and gARMSS, including RAB4B-EGLN2 rs111833532, in RRMS patients.

Predictors	EDSS				MSSS			gARMSS		
	Beta		SE Beta	*p*	Beta	SE Beta	*p*	Beta	SE Beta	*p*
Sex	−0.049		0.043	0.245	−0.058	0.038	0.122	−0.059	0.039	0.136
Disease duration	0.134		0.043	**0.002**	−0.466	0.038	**<0.001**	−0.213	0.04	**<0.001**
Age at onset	0.124		0.043	**0.004**	0.122	0.039	**<0.001**	−0.416	0.04	**<0.001**
RAB4B-EGLN2 rs111833532 (II vs. ID + DD)	0.094		0.043	**0.028**	0.074	0.038	**0.05**	0.093	0.039	**0.018**
HLA-DRB1*15:01 rs3135388 A allele-containing genotypes	0.043	0.043	0.314	0.027	0.038	0.468	0.045	0.039	0.256	

EDSS—Expanded Disability Status Scale; MSSS—Multiple Sclerosis Severity Score; gARMSS—Age-Related Global Multiple Sclerosis Severity; Beta—standardized beta coefficient; SE—standard error.

**Table 4:** Multiple linear regression analysis with predictors of EDSS, MSSS and gARMSS including DYSF-ZNF638 rs10191329 in male PMS patients.

Predictors	EDSS			MSSS			gARMSS		
	Beta	SE Beta	*p*	Beta	SE Beta	*p*	Beta	SE Beta	*p*
Disease duration	0.181	0.113	0.116	−0.491	0.11	**<0.001**	−0.234	0.093	**0.014**
Age at onset	−0.207	0.116	0.08	−0.18	0.113	0.115	−0.699	0.095	**<0.001**
DYSF-ZNF638 rs10191329 CC vs. CA + AA	0.233	0.105	**0.03**	0.168	0.104	0.111	0.18	0.086	**0.04**
HLA-DRB1*15:01 rs3135388 A allele-containing genotypes	−0.199	0.106	0.065	−0.159	0.104	0.131	−0.13	0.087	0.141

EDSS—Expanded Disability Status Scale; MSSS—Multiple Sclerosis Severity Score; gARMSS—Age-Related global Multiple Sclerosis Severity; Beta—standardized beta coefficient; SE—standard error.

**Table 5:** Association of investigated gene variants with circulatory molecular indicators of processes associated with ferroptosis in MS patients.

Product of Lipid Peroxidation	RAB4B-EGLN2 rs111833532 (PMS)		*p*
	II	ID + DD	
4-HNE (pg/mL)	1299.98 ± 370.21	1938.05 ± 1540.60	0.04
	HLA-DRB1*15:01 (MS patients overall)		
	Without allele A	With allele A	
HEL (nmol/L)	12.51 ± 4.03	13.54 ± 3.63	0.018
**Iron metabolism**	**MAP1B rs62363242 (PMS)**		
	GG	GA + AA	
Iron (µmol/L)	15.91 ± 4.14	13.66 ± 4.78	0.03
Transferrin (g/L)	2.27 ± 0.37	2.53 ± 0.44	0.03
Ferritin (ng/mL)	72.25 ± 64.47	58.38 ± 73.62	0.08

Values are presented as mean ± SD; 4-HNE—4-Hydroxynonenal; HEL—hexanoyl-lys adduct; *p*—Mann–Whitney U test.

**Table 6:** Gene variant selection criteria and basic gene/locus information.

GENE or LOCUS	rs ID Number	Allelic Change	Position	MAF	eQTL	RegulomeDB	GWAS	Variants in LD, n
CDKN1A	rs3176326	A/G	Intronic	0.2	Yes	1a	yes	4
					Blood, LCL		CVD	
	rs3176336	A/T	Intronic	0.4	Yes	1f	yes	2
					CD8 T cells, CD4 T cells		CVD	
EGLN2	rs111833532	TCTG/-	Intronic	0.45	Yes	1f	No	None
					Blood, T cells, B cells			
MAP1B	rs62363242	G/A	2.6 kb 3′ of MAP1B	0.34	Yes	1f	No	24
					Artery, brain, heart			
	rs1217817	A/G	13 kb 5′ of MAP1B	0.42	Yes	1f	No	5
					LCL, brain, aorta, CD4+, T cells			
DYSF–ZNF638	rs10191329	C/A	3.9 kb 5′ of DYSF	0.17	Yes for	1f	Yes	2
					ZNF638 in blood, T cells, B cells		MS severity	
MTSS1	rs9643199	A/G	Intronic	0.26	Yes	4	Yes	1
					Brain, blood		MS severity	

MAF—minor allele frequency; eQTL—expression quantitative trait locus; LD—linkage disequilibrium; LCL—lymphoblastoid cell lines.

## Data Availability

The original contributions presented in the study are included in the article; further inquiries can be directed to the corresponding author.

## Abbreviations

The following abbreviations are used in this manuscript:

MS	multiple sclerosis
HLA	human leukocyte antigen
DEG	differentially expressed genes
RR	relapsing–remitting
SP	secondary progressive
P	progressive
*CDKN1A*	cyclin dependent kinase inhibitor 1A
*EGLN2*	egl-9 family hypoxia inducible factor 2
*MAP1B*	microtubule associated protein 1B
eQTL	expression quantitative trait locus
EDSS	Expanded Disability Status Scale
MSSS	Multiple Sclerosis Severity Score
gARMSS	Age-related Global MS Severity Score
CI	confidence interval
N/A	not applicable
SE	standard error
4-HNE	4-Hydroxynonenal
HEL	hexanoyl-lys adduct
MDA	Malondialdehyde
